# Evaluation of Melatonin Secretion and Metabolism Exponents in Patients with Ulcerative and Lymphocytic Colitis

**DOI:** 10.3390/molecules23020272

**Published:** 2018-01-29

**Authors:** Cezary Chojnacki, Janusz Błasiak, Jakub Fichna, Jan Chojnacki, Tomasz Popławski

**Affiliations:** 1 Department of Clinical Nutrition and Gastroenterological Diagnostics, Medical University, 90-647 Lodz, Poland; jan.chojnacki@umed.lodz.pl; 2 Department of Molecular Genetics, University of Lodz, 90-647 Lodz, Poland; janusz.blasiak@biol.uni.lodz.pl (J.B.); tomasz.poplawski@biol.uni.lodz.pl (T.P.); 3 Department of Biochemistry, Medical University of Lodz, 90-647 Lodz, Poland; jakub.fichna@umed.lodz.pl

**Keywords:** melatonin, TPH1, AANAT, ASMT, ulcerative colitis, lymphocytic colitis

## Abstract

Inflammatory bowel diseases, particularly ulcerative colitis (UC) and lymphocytic colitis (LC), affect many people. The role of melatonin in the pathogenesis of UC is precisely determined, whereas in LC it remains unknown. The aim of this study was to compare the expression of the melatonin-synthesizing enzymes tryptophan hydroxylase (TPH1), arylalkylamine-*N*-acetyltransferase (AANAT), and *N*-acetylserotonin methyltransferase (ASMT) in the colonic mucosa and urinary excretion of 6-sulfatoxymelatonin in patients with ulcerative and lymphocytic colitis. The study included 30 healthy subjects (group C), 30 patients with severe ulcerative colitis (group UC), and 30 patients with lymphocytic colitis (group LC). The diagnosis was based on endoscopic, histological, and laboratory examinations. Biopsy specimens were collected from right, transverse, and left parts of the colon. The levels of mRNA expression, TPH1, AANAT, and ASMT were estimated in the colonic mucosa with RT-PCR. The urine concentration of aMT6s was determined by the photometric method. The expression of TPH1, AANAT, and ASMT in colonic mucosa in UC and LC patients was significantly higher than in healthy subjects. Significant differences were found in the urinary aMT6s excretion: group C—13.4 ± 4.8 µg/24 h, group UC—7.8 ± 2.6 µg/24 h (*p* < 0.01), group LC—19.2 ± 6.1 µg/24 h (*p* < 0.01). Moreover, a negative correlation was found between fecal calprotectin and MT6s—in patients with UC − r = −0.888 and with LC − r = −0.658. These results indicate that patients with UC and those with LC may display high levels of melatonin-synthesizing enzymes in their colonic mucosa, which could possibly be related to increased melatonin synthesis as an adaptive antioxidant activity.

## 1. Introduction

Melatonin plays an important role, which is particularly protective, in the gastrointestinal (GI) tract, and the GI tract is its rich source [1,2,3]. Exogenous l-tryptophan, which may be found in many food products, is a substrate for the synthesis of this indoleamine. l-tryptophan is converted to 5-hydroxytryptamine (serotonin) by tryptophan hydroxylase (TPH1) and 5-hydroxytryptophan decarboxylase (DDC). Serotonin is then converted to *N*-acetyltryptamine by arylalkylamine-*N*-acetyltransferase (AANAT), and finally *N*-acetylserotonin methyltransferase (ASMT) conditions the conversion of *N*-acetyltryptamine to *N*-acetyl-5-methoxytryptamine (melatonin) [4,5].

The expression of TPH1, AANAT, and ASMT is regulated by the adrenergic system, and may change under the influence of many agents, including neuromodulatory, hormonal, and inflammatory factors [6,7]. In healthy subjects, melatonin is absorbed from the GI tract and transported by the portal vein system to the liver, where it is metabolized mainly to 6-sulfatoxymelatonin (aMT6s) [8,9]. The 6-sulfatoxymelatonin is excreted with urine, and its content in 24-h urine collection is recognized as a good index of pineal and extra-pineal melatonin synthesis [10,11].

An alternative catabolic pathway in the liver includes melatonin oxidation by indoleamine 2,3-dioxygenase and myeloperoxidase to *N*-acetyl-formylo-5-metoxykynuramine (AFMK) and *N*-acetyl-5-metoxykykuramine (AMK) [12,13].

Melatonin is also metabolized non-enzymatically by free radicals and other oxidants. After neutralizing two hydroxyl radicals (OH·), it is converted to cyclic 3-hydroxymalatonin, or it is metabolized to kynuramine derivatives [14,15]. In some extrahepatic tissues, the process is even more intensive, because at low activity of P450 monooxygenase, only a small amount of aMT6s is generated, and AFMK becomes the main product of melatonin oxidation [16]. The extrahepatic melatonin processes and their rates are still poorly recognized. In addition to these, direct interactions of melatonin with reactive oxygen species may increase the activity of antioxidants and antioxidant enzymes such as superoxide dismutase (SOD), glutathione peroxidase (GPx), and glutathione reductase (GSH), and induce the downregulation of pro-oxidant enzymes such as nitric oxide synthase and lipogenase [17,18,19].

In patients with UC, melatonin synthesis and metabolism depend on many agents, particularly on the nature and severity of the inflammatory process [20].

UC is a chronic disease with periods of exacerbation and remission. The pathogenesis of UC is complex, involving inflammatory and immune factors, endothelial barrier dysfunction, and the overproduction of reactive oxygen forms, all of which play a role in the destruction of colonic mucosa [21,22]. Inflammatory cells such as neutrophils, lymphocytes T and B, monocytes macrophages, and mast cells infiltrate tissues in the region of inflammation. The accumulation of immune cells in the colonic tissue and their activation induces an inflammatory response and leads to the local elevation of pro-inflammatory cytokines [23,24]. The intensity of infiltration with inflammatory cells and the predominance of selected cells depends on the UC stage. Neutrophils predominant in the period of exacerbation build clusters, which are the so-called crypt abscesses. In this phase of the disease, an increase in the number of enterochromaffin cells (EC) could be observed, which was proven in our previous studies [25] and in the reports of other authors [26,27]. It should be expected that when the number of EC cells increases, there will also be an increase of their products, including melatonin.

Lymphocytic colitis (LC) is a common cause of chronic diarrhea, abdominal discomfort, and other gastrointestinal symptoms [28,29]. Its etiology is unknown—infections, immunologic, hormonal, and other factors are suspected [30,31,32,33]. A colonoscopy usually shows normal colonic mucosa, but in microscopic examination, changes are found in biopsies from the right and left part of the colon [34,35]. This disease is mainly characterized by intraepithelial lymphocytosis—more than 20 lymphocytes per 100 surface colonocytes [36,37]. Furthermore, a hypertrophy of colonic neuroendocrine cells was observed [38], but their secretion activity was not estimated.

The aim of this study was to compare the expression of melatonin-synthesizing enzymes (TPH1, AANAT, and ASMT) in colonic mucosa and urinary excretion of aMT6s in patients with UC and LC.

## 2. Results

The initial laboratory tests indicate that inflammatory exponents, such as C-reactive protein and fecal calprotectin, were significantly higher in patients with UC compared to those with LC—*p* < 0.001 (Table 1). The urinary excretion of 6-sulfatoxymelatonin was lower in UC patients than in those with LC—7.86 ± 2.69 µg/24 h vs. 19.2 ± 6.14 µg/24 h (*p* < 0.001, Table 1).

**Figure 1:**
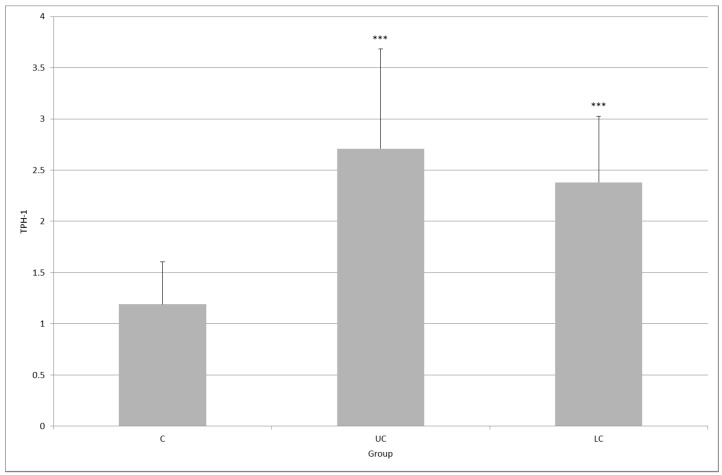
Expression of tryptophan hydroxylase (TPH1) in colonic mucosa in healthy subjects (C) and in patients with ulcerative colitis (UC) and lymphocytic colitis (LC); *** *p* < 0.001, the difference between UC and LC was not statistically significant (*p* = 0.455).

In healthy subjects, TPH1 expression in colonic mucosa was 1.19 ± 0.41; in patients with UC, it was 2.71 ± 0.97 (*p* < 0.001); and in patients with LC, it was 2.38 ± 0.64 (*p* < 0.001, Figure 1). No significant difference was observed between the UC and LC group (*p* = 0.455).

**Figure 2:**
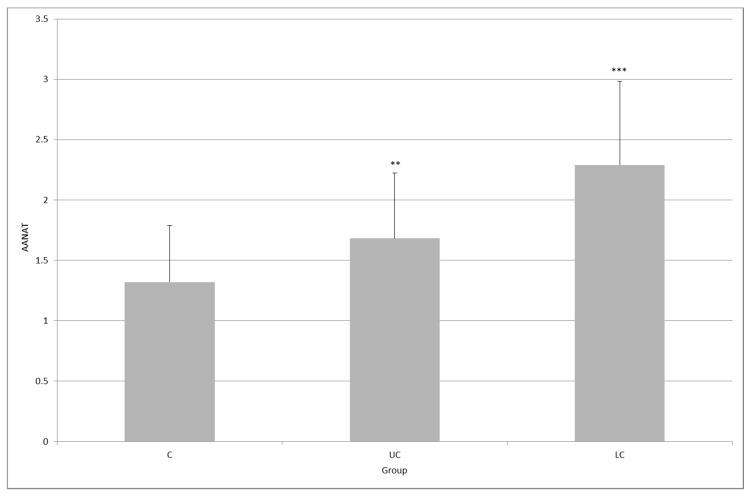
Expression of arylalkylamine-*N*-acetyltransferase (AANAT) in the colonic mucosa of healthy subjects (C), and in patients with ulcerative colitis (UC) and lymphocytic colitis (LC); ** *p* < 0.01, *** *p* < 0.001; the difference between UC and LC was statistically significant (*p* < 0.01).

The level of AANAT expression in the control group was 1.32 ± 0.46, whereas in the UC group it reached 1.68 ± 0.54 (*p* < 0.01), and in the LC group, it was 2.29 ± 0.69 (*p* < 0.001). The difference between inflammatory groups was statistically significant (*p* < 0.01, Figure 2).

**Figure 3:**
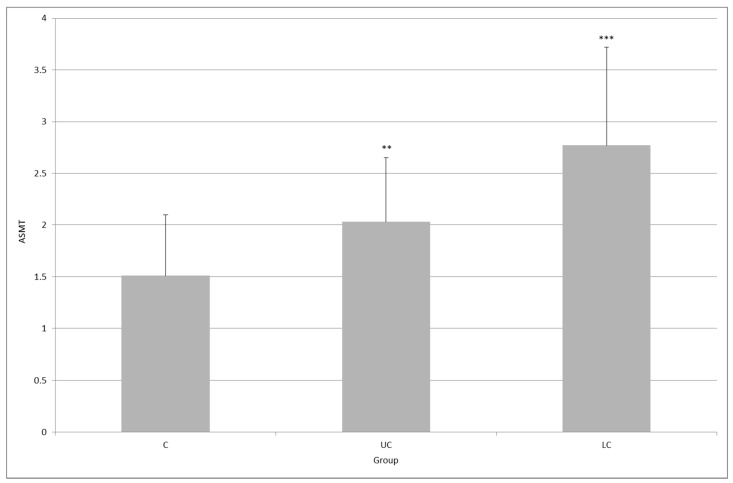
Expression of *N*-acetylserotonin methyltransferase (ASMT) in the colonic mucosa of healthy subjects (C), and in patients with ulcerative colitis (UC) and lymphocytic colitis (LC); ** *p* < 0.01, *** *p* < 0.001; the difference between UC and LC groups was statistically significant (*p* < 0.01).

The level of ASMT expression in both UC and LC groups was higher than that in the control group: 1.51 ± 0.58 versus 2.03 ± 1.97 (*p* < 0.01) and 2.77 ± 2.61 (*p* < 0.001). Here, the difference between the UC group and the LC group was statistically significant (*p* < 0.01, Figure 3). 

**Figure 4:**
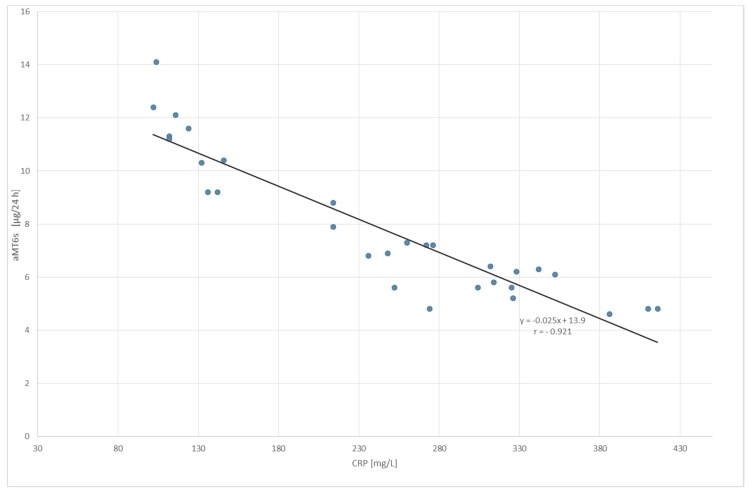
Correlation between the serum level of C-reactive protein (CRP) and urinary 6-sulfatoxymelatonin (aMT6_s_) excretion in patients with ulcerative colitis; *p* < 0.001.

**Figure 5:**
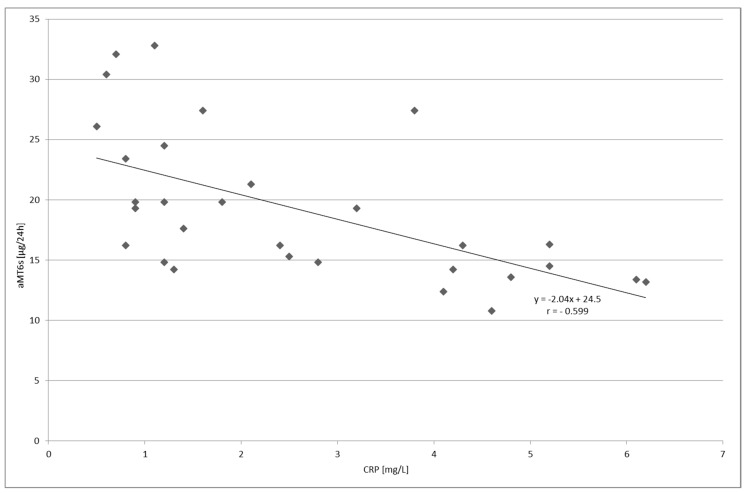
Correlation between the serum level of C-reactive protein (CRP) and urinary 6-sulfatoymelatonin (aMT6s) excretion in patients with lymphocytic colitis, *p* < 0.001.

A negative correlation was found between the serum level of C-reactive protein and urinary aMT6s excretion in the group with UC (*p* < 0.001, Figure 4) as well as in the group with LC (*p* < 0.01, Figure 5).

**Figure 6:**
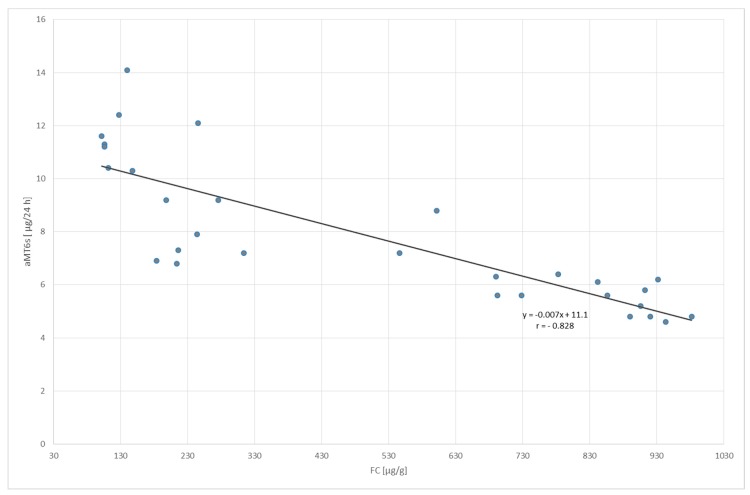
Correlation between fecal calprotectin (FC) concentration and urinary 6-sulfatoxymelatonin (aMT6s) excretion in patients with ulcerative colitis; *p* < 0.001.

**Figure 7:**
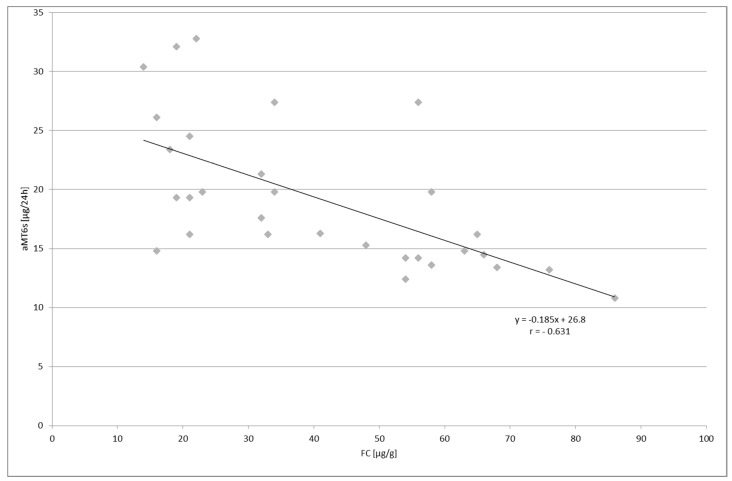
Correlation between fecal calprotectin (FC) concentration and urinary 6-sulfatoxymelatonin (aMT6s) excretion in patients with lymphocytic colitis; *p* < 0.01.

Similarly, a negative correlation was indicated between the volume of fecal calprotectin in both groups; *p* < 0.001 and *p* < 0.01 for UC and LC patients, respectively (Figure 6 and Figure 7).

## 3. Discussion

The obtained results confirm our earlier observations, and indicate that the expression of TPH1 and HIOMT (hydroxyindole-*O*-methyltransferase) in colonic mucosa is significantly higher in UC patients than in healthy subjects [25]. Therefore, it should be acknowledged that inflammatory processes in the colon are accompanied by an increased secretion of enteral melatonin. However, decreased plasma levels of melatonin and urine excretion of aMT6s were observed in patients with ulcerative colitis [39,40]. This may result from the fact that the synthesis and catabolism of melatonin, both in the colon and in the liver, can be modified by many factors, including pro-inflammatory cytokines. Furthermore, melatonin is also metabolized non-enzymatically due to its “consumption” in the reaction with oxygen free radicals at the infection sites.

A particularly important outcome of the study is that now it can be presumed that the level of urinary aMT6s excretion can serve as a biomarker and activity index of colon inflammation in patients with UC, along with C-reactive protein and fecal calprotectin. This may have a tremendous implication for the further diagnosis and treatment efficacy of UC patients.

Unlike in UC, the activity index of LC is poorly determined, and is mainly related to clinical symptoms [41,42,43]. In this disease, the results of the laboratory tests are usually normal or disclose unspecific abnormalities. Patients with LC also have a normal endoscopic appearance, with an occasional erythema and/or patchy edema distributed along the colon [44,45]. The histological picture is a significant lymphocytic infiltration in the surface epithelium, but there are also plasma cells, eosinophils, mast cells, macrophages, and neutrophils with a normal architecture of the crypt [46,47]. Enterochromaffin cells are also present at sites of inflammation. Several potential markers for the diagnosis of LC do exist, but their productive capacity still needs confirmation and measurement in a clinical setting [48]. A markedly higher density of chromogranin A-cells was shown to be present in LC colonic mucosa [49]. Another study not only confirmed these results, but also demonstrated that the significant increase of CgA+ cells in lymphocytic colitis is present both in the right and left colon [50]. The authors suspected that if confirmed, this finding might permit the distinction between the diagnosis of LC and IBS by collecting biopsies during simple sigmoidoscopy. It should be expected that an increase in the number of enterochromaffin cells in colonic mucosa would be also found in patients with UC [26,27] and IBS (Irritable Bowel Syndrome) [51,52], and melatonin can be synthesized in other inflammatory cells, particularly in human lymphoid cells [53]. 

Our study indicates that melatonin-synthesizing enzymes and urine aMT6s excretion were higher in patients with LC than in healthy subjects, which confirms their diagnostic potential. Undisturbed melatonin synthesis arguably protects against the destruction of colonic mucosa due to its antioxidant, anti-inflammatory, and immunoregulatory activity, particularly in T-cells response-based diseases [54,55]. There is still no proof of whether melatonin supplementation may be useful in the complex treatment of patients with LC and with UC [56,57,58], as well as other diseases of digestive tract [59,60], but our findings form a good background for future research in this field.

## 4. Material and methods

### 4.1. Patients

The study included 30 healthy subjects (control group C, aged 38.9 ± 9.4 years), 30 patients with acute phase of ulcerative colitis (group UC, aged 41.4 ± 10.2 years), and 30 patients with lymphocytic (group LC, aged 43.7 ± 12.4 years). The study was performed in the years from 2009 to 2016. The diagnosis was based on clinical, endoscopic, and histological examinations. Only patients with inflammatory changes in whole colon were included in the study. The patients with UC were classified as in the very severe stage according to Mayo Score/Disease Activity Index for Ulcerative Colitis. The patients with LC had normal endoscopic pictures, but their intraepithelial lymphocytes went over 25 per 100 epithelial cells. Moreover, the patients had intensive and chronic symptoms such as non-bloody diarrhea, abdominal pain, fecal incontinence, and others.

Exclusion criteria: small intestinal bacterial overgrowth, food intolerance, parasitosis, exocrine pancreatic deficiency, thyroid dysfunction, metabolic and mental diseases, and chronic use of drugs, except for 5-aminosalicylates.

### 4.2. Study Design and Procedures

The following routine laboratory tests were performed in all of the subjects: blood cells count; quantification of protein, glucose, bilirubin, iron, urea, creatinine, and thyroglobulin concentration; and activity of alanine and asparagine aminotransferase, alkaline phosphatase, gamma-glutamyl transpeptidase, amylase, and lipase.

Furthermore, tests were also performed for serum concentrations of C-reactive protein (by latex agglutination photometric assay—COBAS INTEGRA 800, Indianapolis, IN, USA), fecal calprotectin (Sandwich ELISA—Quantum Blue Reader) and urine concentrations of 6-sulfatoxymelatonin (aMT6s) with enzyme immunoassay.

On the day of testing urinary aMT6s excretion, the subjects received only condensed liquid meals (Nutridrink 3 × 400 mL) with a total energy value of 1800 kcal, and 1500 mL noncarbonated isotonic mineral water. After completion of 24-h urine collection, the urine was centrifuged, and the samples were stored at −70 °C. The urinary aMT6s concentration was determined using Immuno Biochemical Laboratories kit (No. RE 54031). The measurements were performed by photometry at a wavelength of 450 nm (Expert 96-Reader—Biogenet, Józefów, Poland). The obtained results were converted from nanogram per mililiter to microgram/24 h.

Biopsy specimens were collected from the right, transverse, and left colon. The level of mRNA expression was estimated with RT-PCR, and 50 mg of colonic tissues were used for this purpose. Briefly, colonic tissues were rapidly permeated to stabilize and protect cellular RNA with RNA stabilization reagent RNAlater^®^ (Quiagen, Hilden, Germany). Prior to total RNA isolation, colonic tissues were homogenized with TissueRuptor (Quiagen, Hilden, Germany). Next, the total RNA was isolated using Quiagen RNeasy Plus Mini Kit (Quiagen, Hilden, Germany), according to the manufacturer’s protocol. The quantity and quality of isolated RNA were estimated spectrophotometrically by Take3 plate on Synergy HT Microplate Reader (BioTek Instruments, Winooski, VT, USA). The real-time gene expression analysis was performed using the TaqMan Gene Expression Assays (Thermo Fisher Scientific, Waltham, MA, USA) with probes for TPH1, (Assay ID: Hs00188220_m1), AANAT (Assay ID: Hs01063208_g1), and ASMT (Assay ID: Hs00187839_m1), and a SensiFASTTM Probe No-ROX One-Step Kit (Bioline, Taunton, MA, USA). The HPRT (The hypoxanthine phosphoribosyltransferase, Assay ID: Hs01003267_m1) gene was a reference. Real-time PCR reaction was performed with BioRad CFX96 (BioRad, Hercules, CA, USA), according to the suggested RT-qPCR conditions. Expression analysis was performed with CFX Manager 1.6 software (BioRad, Hercules, CA, USA) using ΔΔC_t_ method with the HPRT gene as the reference target.

### 4.3. Ethics

The study was conducted in accordance with the Declaration of Helsinki and the principles of Good Clinical Practice. Written consent was obtained from each subject enrolled in the study, and the study protocol was approved by the Bioethics Committee of the Medical University of Lodz (RNN/242/06/KB).

### 4.4. Statistical Analysis

The non-parametric Kruskal–Wallis test was used to evaluate the expression of TPH1, AANAT and ASMT, and urinary aMT6s excretion in three groups: C, UC, and LC. The Mann–Whitney test was used for the comparison of mean values. The correlation between the value of urinary aMT6s excretion and the concentration of plasma CRP and fecal calprotectin was estimated by determination of Pearson’s correlation coefficient, a linear regression equation, and the rang Spearman coefficient. The differences between the results were regarded as significant when a *p* = 0.05–0.001. Statistica 9.0 (StatSoft, Inc., Palo Alto, CA, USA) and MS Excel @007 (Microsoft Co., Redmond, WA, USA) were used for statistical analysis.

## 5. Conclusions

These results indicate that patients with ulcerative colitis and those with lymphocytic colitis may display high levels of melatonin-synthesizing enzymes in their colonic mucosa, which is possibly related to the increased melatonin synthesis as adaptive antioxidant activity. The high synthesis of melatonin in LC possibly protects colonic mucosa against macroscopic damage.

## Figures and Tables

**Table 1:** General characteristics of subjects included in the study and some initial laboratory results; C-reactive protein (CRP), fecal calprotectin (FC), urinary 6-sulfatoxymelatonin excretion (aMT6_s_).

Features		Control Group (*n* = 30)	Ulcerative Colitis (*n* = 30)	Lymphocytic Colitis (*n* = 30)
Age—years		38.9 ± 9.4	41.4 ± 10.2	43.7 ± 12.4
Gender	M	16	13	14
	K	14	17	16
CRP (mg/L)		1.97 ± 1.05	242.9 ± 99.4	2.58 ± 1.81
FC (µ/g)		25.8 ± 9.72	498.6 ± 338.3	40.8 ± 21.0
aMT6_s_ (µg/24 h)		13.4 ± 4.87	7.86 ± 2.69	19.2 ± 6.14
